# Downregulation of miR-29a as a possible diagnostic biomarker for schizophrenia

**DOI:** 10.1007/s11033-024-09428-2

**Published:** 2024-05-05

**Authors:** Parya Alizadeh Khosroshahi, Hamidreza Ashayeri, Mohammad Ghanbari, Ayyoub Malek, Sara Farhang, Mehdi Haghi

**Affiliations:** 1 Department of Animal Biology, Faculty of Natural Sciences, University of Tabriz, Tabriz, Iran; 2 Student Research Committee, Tabriz University of Medical Sciences, Tabriz, Iran; 3 Clinical Research Development Center, Mohammad Kermanshahi and Farabi Hospitals, Imam Khomeini, Kermanshah University of Medical Sciences, Kermanshah, Iran; 4 University Medical Center Groningen, University of Groningen, University Center for Psychiatry, Groningen, The Netherlands; 5 Research center of psychiatry and behavioral sciences, Tabriz university of medical sciences, Tabriz, Iran

**Keywords:** Schizophrenia, Epigenetics, miR-29a, Non-coding RNA, Biomarker, Real-time PCR

## Abstract

**Background:**

MicroRNAs (miRNAs) are epigenetic factors regulating many genes involved in brain development. Dysregulation of miRNA could result in dysregulation of genes which may contribute to diseases affecting the brain and behavior (e.g., schizophrenia). miR-29 family is a miRNA family contributing to brain maturation. miR-29 knockout in animal studies is reported to correlate with psychiatric disorders very similar to those seen in schizophrenia. In this study, we aimed to evaluate the miR-29a level in patients with schizophrenia and its potential value in the diagnosis of schizophrenia.

**Materials and methods:**

The serum sample of 42 patients with schizophrenia and 40 healthy subjects were obtained from the Azeri Recent onset/Acute phase psychosis Survey (ARAS) Cohort study. After preparations, the expression level of miR-29a was investigated by real-time PCR. The SPSS and GraphPad prism software were used to analyze the relation between miR-29a level and clinical parameters and its potential as a biomarker for the diagnosis of schizophrenia.

**Results:**

Our study showed a significantly lower miR-29a level in patients compared to healthy controls (*p* = 0.0012). Furthermore, miR-29a level was significantly lower in some types of schizophrenia (*p* = 0.024). miR-29a level was not related to sex, age, or heredity (*p* > 0.05). miR-29a also showed 80% specificity and 71.43% sensitivity in the diagnosis of schizophrenia.

**Conclusion:**

Downregulation of miR-29a in schizophrenia is significantly related to the development of this illness. It might have the potential as a biomarker for schizophrenia.

## Introduction

Schizophrenia affects thinking, behaviors, and feelings. The prevalence is around 1% of the population [1] but it is one of the mental illnesses capable of disrupting lives of patients and people around them. The diagnosis of schizophrenia includes a spectrum of symptoms that are classified into two categories: positive and negative symptoms [2]. Both genetic and environmental factors play major roles in the pathogenesis of schizophrenia. According to monozygotic and dizygotic twin studies, the inheritance rate of schizophrenia is estimated to be approximately 97% indicating the importance of genes in the incidence of this disease [3]. Environmental factors like childhood experiences [4], migration [5], and cannabis use [6] also increase the chance of schizophrenia. Increased dopamine levels play a role in the pathophysiology of schizophrenia and drugs that antagonize D2 receptors reveal some of the symptoms [7, 8]. Despite all current treatment options, 10 to 30% of patients show resistance to pharmacologic treatments [9]. These imply that other mechanisms might be involved and a better understanding of the pathogenesis and pathophysiology of disorder is needed for the early detection and development of effective therapeutic strategies.

Environment affects gene expression and probability of schizophrenia increases if a person with high genetic risk is exposed to environmental risk factors [10]. Epigenetic mechanisms explain the interaction between genetics and the environment at a molecular level. Many studies in the field of epigenetics have focused on microRNAs (miRNAs) due to their important mechanism in the post-transcriptional regulation of genes and their effect on the development of many diseases without any change of DNA sequence. Also, there is a reciprocal action between miRNAs and other epigenetic pathways such as DNA methylation which makes miRNA-epigenetic feedback loop in physiological processes [11]. The mature miRNAs are small noncoding RNA molecules (19–23 nucleotides length) that interact with RNA-inducing silencing complex (RISC) and are able to suppress numerous genes via attaching to 3’ UTR region of mRNAs. This leads to the prevention of mRNA translation or mRNA breakage via the enzymatic activity of RISC [12]. Also, many miRNAs show tissue-specific expression patterns and are released from normal or abnormal cells via extracellular vesicles into circulation. Change in the level of specific miRNAs in body fluids may be related to the abnormality of specific cells or organs in the body [13].

Many of these miRNAs regulate genes which play a vital role in the pathophysiology of the central nervous system thereby; dysregulation of these miRNAs can be the origin of abnormal pathways in the development of mental disorders such as schizophrenia [14]. For example, miR-9 and miR-4467 regulate the genes that engage in the regulation of neurotrophin signaling, neuronal differentiation, and nervous system development. Downregulation of miR-9 and upregulation of miR-4467 show a potential diagnostic biomarker in the blood of patients with schizophrenia [15]. miR-29 family are highly noticed because they correlate with the late stage of brain maturation. The role of miR-29 dysregulation is revealed in several neurodegenerative diseases such as Alzheimer’s [16], Huntington’s, ataxia phenotype [17], and Parkinson’s disease [18]. Also, miR-29 knockout in mice is associated with psychiatric disorders very similar to those seen in autism, epilepsy, and schizophrenia [19]. The expression pattern and diagnostic biomarker potential of miR-29 in schizophrenia patients’ serum is not clear yet. However, it is essential to evaluate miR-29a expression pattern and biomarker potential role in patients with schizophrenia due to the crucial role of miR-29a in neuronal differentiation, neurological recovery, axon branching, neural cell survival, and synaptic plasticity [17, 20–23]. The aim of this study was to evaluate miR-29a expression level in the serum of schizophrenia patients compared to normal ones. In this research, we also evaluated the potential biomarker role of miR-29a in patients with schizophrenia.

## Materials and methods

### Sample preparation

This study was performed within the framework of ARAS study. The Azeri Recent onset/Acute phase psychosis Survey (ARAS) [24] is a prospective cohort of Iranian patients with first episode psychosis. This study meets international ethical standards, including the Declaration of Helsinki, and was approved by the Iranian national ethical committee (IR.NIMAD.REC.1396.101). Patients diagnosed with schizophrenia based on Diagnostic and Statistical Manual of Mental Disorders-5 (DSM-5) criteria were enrolled. They were drug naïve or within the first month of starting the medication. After obtaining written informed consent from patients and their legal caregivers, the blood samples of 42 patients with schizophrenia and 40 controls were prepared from Razi hospital (Tabriz, Iran) and immediately stored at -80℃. The control sample involved students or staff of the hospital volunteered to give blood sample for research purpose. Beside the self-report of medical and psychiatric history, a clinical interview evaluated them for major psychiatric disorders and the samples were used if there was no psychiatric condition reported or detected.

### RNA isolation

Total RNA was isolated from serum specimens by using trizol in room temperature based on the manufacturer’s protocol (ziaviz, Iran). The quantity of RNA was assessed by a nanodrop spectrophotometer (Thermo Fisher Scientific, Waltham, USA). RNA samples were stored at -80˚C until complementary DNA (cDNA) synthesis. According to manufacturer’s instructions, approximately 250 µg of total RNA was used as template for cDNA synthesis using add script cDNA synthesis kit (add bio, Korea). For specific cDNA synthesis from target miR-29a and U6 mRNA, specific stem-loop primers were designed which have been illustrated in Table 1. U6 was used as the housekeeping gene for data normalization.

**Table 1:** designed RT-PCR primer sequences

Primer	MiR-29a	U6
Stem-loop primer	5’-GGTCGTATGCAGAGCAGGGTCCGAGGTATCCATCGCACGCATCGCACTGCATACGACCCACTGGTAACCG − 3’	5’- GTCGTATCCAGTGCAGGGTCCGAGGTATTCGCACTGGATACGACAAAAATAT-3’
Forward primer	5’- AACACGCTAGCACCATCTGAA − 3’	5’-GCTTCGGCAGCACATATACTAAAAT -3’
Reverse primer	5’- GGTCGTATGCAGAGCAGGG − 3’	5’- CGCTTCACGAATTTGCGTGTCAT-3’

### Quantitative real-time PCR (qRT-PCR)

For qRT-PCR, specific primers were designed which have been detailed in Table 1. qRT-PCR was performed by using SYBR green MasterMix (Amplicon, Odense, Denmark) on a Light Cycler® 96 Real-Time PCR system (Roche Molecular Systems, Pleasanton, USA). A total volume of 10 µl containing 5 µl MasterMix, 0.3 µl of forward and reverse primers for miR-29a and 0.5 µl of forward and reverse primers for U6, 2.4 µl DNase/RNase free H2O for miR-29a and 2 µl DNase/RNase free H2O for U6, and 2 µl of cDNA was used in each reaction. Amplification was performed with the following settings: 15 min at 95˚C, followed by 45 cycles of 30 s at 95˚C, 30 s at 60˚C and 30 s at 72˚C. All reactions were repeated three times and miR-29a relative expression was evaluated by using the comparative cycle threshold (Ct) method. Then, miR-29a expression levels were normalized to U6 expression levels and difference between miR-29a and U6 Ct values (ΔCt) was calculated for each sample. Finally, miR-29a expression levels in patients and control people were determined by calculating 2 ^−∆Ct^.

### Statistical analysis

SPSS statistics version 26 and GraphPad Prism 9 software were used for statistical analysis. Non-parametric Mann-Whitney and Kruskal Wallis tests were performed to examine expression level and association between miR-29a expression and clinical characteristics of patients with schizophrenia. Receiver operation curve (ROC) was used for evaluating sensitivity and specificity of miR-29a as a potential diagnostic biomarker. The 95% confidence interval (CI) was used and p-values less than 0.05 were considered as significant.

## Results

### Patient characteristics

A total of 82 subjects (42 patients with schizophrenia and 40 healthy individuals) were included in this research. Thirty (71.43%) patients were male and twelve (28.57%) were female and in healthy control individuals 20 were male and 20 were female. The mean age for patients was 31 (SD = ± 13.85 years) and for healthy individuals was 26.4 (SD = ± 6.1 years). Based on DSM-IV schizophrenia classification, approximately 23 patients (54.76%) had paranoid type, 9 patients (21.43%) had disorganized type, and 10 patients (23.81%) had undifferentiated type of schizophrenia. In terms of the familial background, 8 patients (19%) had heredity and 34 patients (81%) had non-heredity form of schizophrenia (Table 2.). While 15% of patients was taking no medication, the majority of them were receiving antipsychotic treatment at the time of assessment: risperidone (42.5%), olanzapine (22.5%), quetiapine (5%) aripiprazole (7.5%) and haloperidol (7.5%).

**Table 2:** Association between miR-29a expression and clinical parameters in patients with schizophrenia

Variable	Number of patients	MiR-29a mean expression (± SE) in tumor samples	Statistical significance	P-value	
Age (years)					
> 26	21	7.522 (0.475)	NS	0.505	
< 26	21	8.003 (0.002)			
**Gender**					
Male	30	8.113 (0.405)	NS	0.079	
Female	12	6.886 (0.682)			
**Inheritance**					
Yes	8	8.623 (1.000)	NS	0.179	
No	34	7.560 (0.371)			
**Disease type**					
Paranoid	23	6.988 (0.468)			
Disorganized Undifferentiated	9 10	9.182 (0.643) 9.364 (0.510)	S	0.024	

NS = Not significant, S = Significant

### The expression of miR-29 in serums of patients with schizophrenia

**Fig. 1:**
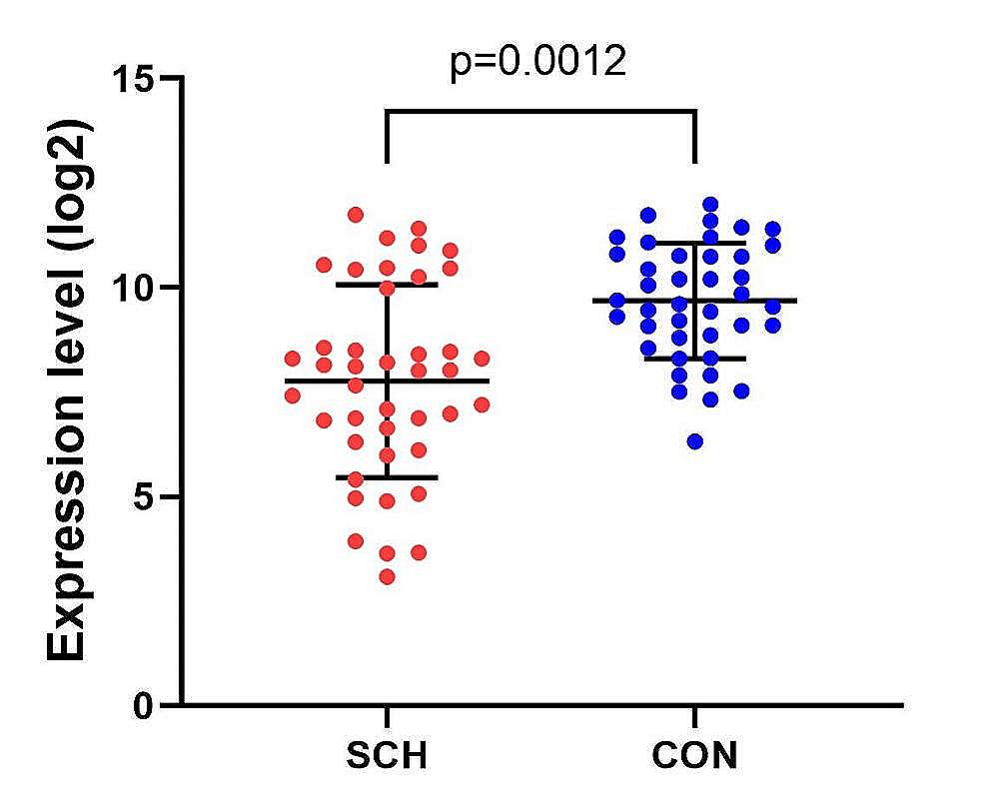
Expression of miR-29a in serum of patients with schizophrenia compared to healthy subjects. CON: healthy controls, SCH: schizophrenia

The expression level of miR-29a in serum of patients with schizophrenia and healthy individuals were assessed by qRT-PCR. Our results revealed that the expression level of miRNA-29a is significantly decreased in serum of schizophrenic patients compared to serum of healthy individuals (p-value = 0.0012) (Fig. 1).

### Association between miR-29a expression levels and clinical parameters

Our results demonstrated that there was a significant association between miR-29a expression level and the types of schizophrenia so that, the expression of miR-29a significantly decreased in serum samples of paranoid type compared to disorganized and undifferentiated types of schizophrenia (p-value = 0.024). However, there was no significant association between miR-29a expression and other clinical features including age, gender, and inheritance (*p* > 0.05).

### The serum expression level of miR-29a as a potential biomarker of schizophrenia

**Fig. 2:**
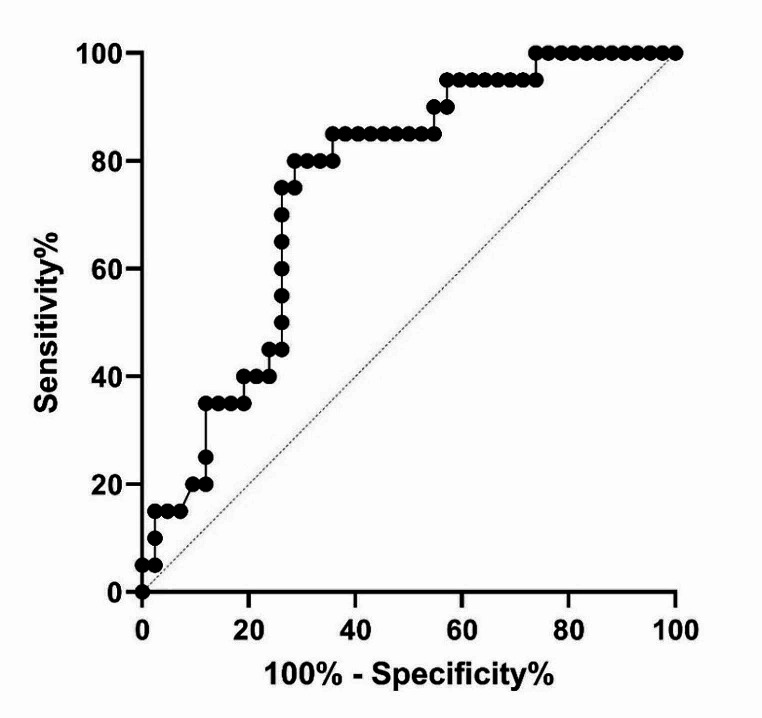
The ROC curve analysis showed a sensitivity and specificity of 80% and 71.43%, respectively. The AUC is 0.7506. AUC: area under the curve, ROC: receiver operation curve

The ROC analysis was performed to evaluate miR-29a potentials as a diagnostic biomarker for schizophrenia. Our result revealed a sensitivity and specificity of 80% and 71.43%, respectively (p-value = 0.0015). The area under curve (AUC) was 0.7506 (Fig. 2. and Table 3).

**Table 3:** The statistical analysis of the ROC curve for miR-29a in patients with schizophrenia

The ROC curve data	Values
The area under the ROC curve	0.7506
Sensitivity (%)	80
Specificity (%)	71.43
Cutoff score	> 367.8
Std. error	0.06263
95% confidence interval	0.6278–0.873
P-value	0.0015
Number of patients with schizophrenia	42
Number of healthy individuals	40

## Discussion

In present study, we revealed significant downregulation of miR-29a in serum of patients with schizophrenia compared to healthy controls (p-value = 0.0012). This miR-29a downregulation was significantly correlated with paranoid type than other main types including disorganized and undifferentiated forms of disease (p-value = 0.024). Also, we concluded that low level of miR-29a in serum can be used as a potential biomarker in diagnosis of schizophrenia (sensitivity = 80% and specificity = 71.43%). Our findings are consistent with previous study by perkins et al. (2007) in which they showed downregulation of 15 miRNAs including miR-29a and miR-29c in postmortem prefrontal cortex tissue study of 15 individuals with schizophrenia (*n* = 13) or schizoaffective disorder (*n* = 2) compared to 21 unaffected individuals (patients miR-29a level was 0.82 fold the unaffected subjects miR-29a level) [25]. Another study by Akif Camkurt et al. (2016) investigated miR-29a-3p serum levels between 16 schizophrenic patients and 16 normal controls report a significantly higher level of miR-29a-3p in patients compared to normal subjects (p-value < 0.001) [26].

Several target genes and molecular pathways of miR-29 are known in brain disorders, which indicate the important role of miR-29 in the brain development. miR-29 family includes miR-29a, miR-29b-1, miR-29b-2 and miR-29c [19]. The mature sequences of these miRNAs are highly conserved in human, mouse and rat. This indicate their common mechanisms in regulation of genes which have crucial role in neurodegeneration and neuronal survival [27]. The mechanism in which miR-29a dysregulation is associated with schizophrenia is not known and need to deep molecular studies. However, recent researches highlight several molecular pathways of miR-29 in brain maturation and neurofunctional disorders. Shioya et al. demonstrated that miR-29 regulates navigator 3 (NAV3) gene which is a regulator of axon guidance. Downregulation of miR-29a is associated with increased level of NAV3 in Alzheimer disease brains [28]. Since NAV3 has is part of neural navigator gene family like NAV1 and NAV 2, dysregulation in NAV 3 expression can lead to abnormal neural growth and play a role is disease such as autism spectrum disorders [29] or Alzheimer disease.

Downregulation of miR-29a is also associated with neural cell death [17]. Roshan et al. revealed that in Spinocerebellar ataxias 17, downregulation of miR-29a and miR-29b lead to elevation of their target genes; β-secretase 1 (BACE1), p53 upregulated modulator of apoptosis (PUMA), and BAK which result in cytochrome releasing and neuronal apoptosis [30]. It is demonstrated that miR-29 by targeting PTEN expression stimulates AKT signaling pathway which has an important role in neural stem/progenitor cells (NSPCs) selfrenewing during brain damage [31]. Ling Yang et al. revealed that in Parkinson’s disease, miR-29a by targeting of mitochondrial antiviral signaling protein (MAVS) prevents MPP+-induced cell death and inflammation. Therefore, miR-29a has neuroprotective role against inflammation and oxidative stress which are common feature of neuropsychiatric disorders such as schizophrenia, autism, and depression [32]. A recent research that carried out in mice model, revealed DNMT3A as a direct target of miR-29, has an important role in postnatal mammalian brain development and its upregulation during knock out of miR-29 associated with neurobehavioral sequelae as seen in autism spectrum disorders, epilepsy, and schizophrenia [19]. DNA methyltransferase DNMT3A is responsible for de novo non-canonical CH (non-CG where H = A, C, T) methylation which is increased in the postnatal brain at birth and then severely decreased several weeks later [33]. Regulation of DNMT3A depends on miR-29 expression pattern in postnatal brain during lifespan so that downregulation of miR-29 associated with increased CH methylation in genes such as CHL1, FZD3, and SOX5 which are associated with schizophrenia [19]. A Meta-analysis has shown that synaptophysin levels reduce in prefrontal cortical cortex and hippocampus of schizophrenic patients [34]. Postmortem studies and genetic evidences show a reduced synaptic plasticity in schizophrenia [35]. Therefore, miR-29 family regulate neuronal activity and synaptic function during lifespan. Downregulation of miR-29 in serum of patients can cause impairment in neural and synaptic activity in schizophrenia. However precise mechanisms and molecular pathways of miR-29 downregulation in schizophrenia need to more studies.

There are very limited reports about patients with psychotic deriders from the Middle east, including Iran. The main strength of this study was the nature of the sample, standard diagnostic tools and the timing of blood sampling. However, results might be limited with the cross-sectional design, and using blood samples. For future studies we recommend using larger sample size and including further clinical outcome to determine value of microRNA levels as a screening tool, considering effect of treatment on microRNA levels, and using lumbar puncture samples for a more accurate investigation of conditions which effect brain.

## Conclusion

In conclusion, results of this study showed that miR-29a is significantly downregulated in serums of patients with schizophrenia compared to healthy individuals. Also, low level of miR-29a in serum of schizophrenic patients was significantly associated with paranoid type of schizophrenia. According to ROC analysis, we concluded that miR-29a may be considered as a potential diagnostic biomarker in patients with schizophrenia.

## Data Availability

The data that support the findings of this study are available from the corresponding authors, Sara Farhang or Mehdi Haghi, upon reasonable request.

## Abbreviations

ARAS: Azeri Recent onset/Acute phase psychosis Survey
AUC: area under curve
BACE1: β-secretase 1
cDNA: Complementary DNA
CI: confidence interval
MAVS: mitochondrial antiviral signalling protein
mRNA: messenger RNA
miRNA: microRNA
NSPC: neural stem/progenitor cell
PUMA: p53 upregulated modulator of apoptosis **qRT-PCR**:Qualitative real-time polymerase chain reaction
ROC: Receiver operation curve
